# Fairness and objectivity of a multiple scenario objective structured clinical examination

**DOI:** 10.3205/zma001234

**Published:** 2019-05-16

**Authors:** Johannes Spanke, Christina Raus, Annekathrin Haase, Aniela Angelow, Fabian Ludwig, Gesine Weckmann, Carsten Oliver Schmidt, Jean-Francois Chenot

**Affiliations:** 1 University Medicine Greifswald, Institute for Community Medicine, Department of General Practice and Family Medicine, Greifswald, Germany; 2 European University of Applied Sciences, Faculty of Applied Health Sciences, Rostock, Germany; 3 University Medicine Greifswald, Institute for Community Medicine, SHIP-KEF, Greifswald, Germany

**Keywords:** medical students, medical education, objective structured clinical examination, rater effects

## Abstract

**Introduction: **The aim of the Objective Structured Clinical Examination (OSCE) is a standardized and fair assessment of clinical skills. Observing second clinical year medical students during a summative OSCE assessing a General Practice clerkship, we noticed that information exchange with peers led to a progressively faster and overly focused management of simulations. Therefore, we established a Multiple Scenario-OSCE (MS-OSCE) where all students had to manage the same chief complaint at a station but it’s underlying scenarios being randomly changed during students’ rotation through their parcours. We wanted to ensure they fully explore differential diagnosis instead of managing their task influenced by shared information. We wanted to assess if a MS-OSCE violates the assumption of objectivity and fairness given that students are not tested with the same scenarios.

**Methods: **We developed and piloted five OSCE stations (chest pain, abdominal pain, back pain, fatigue and acute cough) with two or three different underlying scenarios each. At each station these scenarios randomly changed from student to student. Performance was assessed with a checklist and global rating. The effect of scenarios and raters on students’ grades was assessed calculating the intraclass correlation coefficient with a fixed effect two level linear model.

**Results: **A total of 169 students and 23 raters participated in the MS-OSCE. The internal consistency over all stations was 0.65 by Cronbach’s alpha. The difference of the mean grades between the scenarios of a given chief complaint ranged from 0.03 to 0.4 on a 1 to 5 grading scale. The effect of scenarios on the variance of the final grades at each station ranged from 4% to 9% and of raters from 20% to 50% when adjusted for students’ skills.

**Conclusions: **The effect of different scenarios on the grades was relevant but small compared to the effect of raters on grades. Improving rater training is more important to ensure objectivity and fairness of MS-OSCE than providing the same scenario to all students.

## Zusammenfassung

**Hintergrund: **Das Ziel einer Objective Structured Clinical Examination (OSCE-Prüfung) ist eine standardisierte und faire Prüfung klinischer Fertigkeiten. Nach dem Blockpraktikum Allgemeinmedizin im 2. klinischen Jahr (4. Studienjahr) werden die Studierenden mit einer OSCE-Prüfung Allgemeinmedizin an Simulationspatienten beurteilt. In der Vergangenheit konnten wir beobachten, dass prüfungsrelevante Informationen während der Prüfung unter den Studierenden ausgetauscht wurden. Dies führte zu einer zunehmend hastigen und unpräzisen Interaktion mit dem Simulationspatienten. Daher entwickelten wir eine Multiple-Scenario-OSCE-Prüfung (MS-OSCE), bei der an jeder Station einem bestimmten Beratungsanlass unterschiedliche Szenarios zugrunde gelegt werden, die bei gleichlautender Aufgabenstellung während der Rotation einer Studierendengruppe innerhalb jeder Station randomisiert gewechselt wurden. Eine MS-OSCE soll die Studierenden veranlassen, mögliche Differentialdiagnosen gründlicher zu explorieren, anstatt ihre Aufgaben unter dem Einfluss von weitergeleiteten Informationen vorangehender Prüfungskandidaten zu lösen. Wir wollten beurteilen, ob die unterschiedlichen Szenarien einer Station vergleichbare Schwierigkeiten aufwiesen und welche Faktoren die Fairness und Objektivität der MS-OSCE beeinflussen.

**Methoden:** Wir entwickelten und pilotierten fünf OSCE-Stationen (Beratungsanlässe: Brustschmerz, Bauchschmerz, Rückenschmerz, Müdigkeit und akuter Husten) mit zwei oder drei unterschiedlichen Szenarien für den an der jeweiligen Station vorgesehenen Beratungsanlass. Der Wechsel der Szenarios an jeder Station erfolgte randomisiert von Student/in zu Student/in. Die Leistungsbewertung der Studierenden erfolgte sowohl mit einer Checkliste als auch mit einem globalen Rating. Der Effekt der Szenarien und der Prüfer/-in auf die Noten der Studierenden wurde durch Berechnung des Intraclass-Korrelationskoeffizienten mit einem linearen Zweiebenen-Modell mit fixen Effekten ermittelt.

**Ergebnisse: **An der MS-OSCE nahmen insgesamt 169 Studierende und 23 Prüfer/innen teil. Die mittels Cronbach’s alpha berechnete Interne Konsistenz über alle Stationen auf einer Notenskala von 1 bis 5 betrug 0,65. Die mittlere Notendifferenz zwischen den Szenarien eines Beratungsanlasses reichte von 0,03 bis 0,4. Der Einfluss der Szenarien auf die Varianz der durchschnittlichen Noten pro Station lag nach Adjustierung für die Fähigkeiten der Studierenden bei 4% bis 9%. Der Einfluss der Prüfer/-innen reichte von 20% bis 50%.

**Schlussfolgerung: **Der Einfluss der unterschiedlichen Szenarien einer Station auf die Note war gering im Vergleich zum Einfluss der Prüfer/-in. Um die Objektivität einer MS-OSCE zu gewährleisten muss eine adäquate Prüferschulung erfolgen. Verbesserung der Interrater-Reliabilität ist wichtiger für Fairness und Objektivität, als alle Studierenden mit demselben Szenario zu prüfen.

## Introduction

The Objective Structured Clinical Examination (OSCE) is a common method to assess clinical and procedural skills in undergraduate medical education since its introduction by Harden et al. in 1975 [1]. We assess the clerkship in General Practice of second clinical year medical students with a summative OSCE. Standardized patients (SP) are used in OSCEs to ensure that each student encounters identically portrayed scenarios [2], [3]. As inherent to any assessment of clinical competence, objectivity (i.e. validity, reliability, efficiency, transparency) is susceptible to implementation and realisation imperfections [4], [5], [6], [7]. Additionally, cheating during OSCEs poses a threat to objectivity and fairness [8], [9], [10]. Fairness is the quality of making judgements that are free from bias and discrimination and requires conformity rules and standards for all students [11]. 

We assume that exchange of detailed information about the content of the OSCE-stations might be the cause for observations we made in previous years: It takes three days to assess the entire cohort of second clinical year medical students. We noticed that many students scheduled after the first round managed OSCE-simulations progressively faster and disproportionally focused. They often jumped to conclusions based on information they did not elicit during the simulation. For example, they made diagnosis and management decisions without having completed physical examination and history taking. As “communication skills” on electronic platforms are common among modern-day students, the sharing of information about the content of exams has become easier [12], [13]. We identified internet blogs from medical students who finished the OSCE, providing hints to other students. We observed that students used case-specific information during ongoing examinations. Although several studies found that this kind of cheating does not necessarily effect test results to a relevant extent [9], [10], [14], [15], we believe this had a negative effect on the performance of students during examination.

Therefore, we established a Multiple Scenario-OSCE (MS-OSCE) where all students had to manage the same chief complaint with different underlying scenarios. The goal of multiple scenarios is to ensure that all students take a thorough history and perform a complete physical examination to explore the differential diagnoses at each OSCE-station, despite prior information received from students who already completed the OSCE. Varying an OSCE station while students are rotating on their examination parcours seems to be frequently done but has not been published extensively, whereas the effects of changing raters during an examination is well documented [16].

The aim of our analysis was to asses if a MS-OSCE violates the assumption of objectivity and fairness, given that all students are not tested with identical scenarios.

Our hypothesis is that testing the management of a chief complaint with multiple scenarios does not unfairly affect the grading of students’ performance.

## Methods

This is an observational study about the implementation of the MS-OSCE concept to assess the General Practice clerkship of 169 second clinical year medical students (58% female, median age 26 years, range 22 to 37) [17]. Two students dropped out due to sickness.

### Development of the MS-OSCE stations

In accordance with the competencies and learning objectives of the General Practice curriculum we generated an OSCE blueprint and developed five OSCE- stations, each testing one chief complaint with two to three different scenarios. Chief complaints for the OSCE were published on the website of the department of General Practice four weeks in advance to the OSCE to allow students to prepare for the examination. Chief complaints were: chest pain, abdominal pain, back pain, fatigue and acute cough. There are national guidelines for managing these complaints except for abdominal pain. The chief complaints with the respective underlying scenarios are summarized in table 1 (Tab. 1). The multiple scenarios chest pain station had been piloted in the previous year OSCE. The other OSCE-stations have been piloted with volunteer students.

#### Simulation patients and rater training

The scenarios for each chief complaint were standardized. Theatre students and lay-actors were recruited as simulation patients (SP). SPs were instructed to use a standardized opening phrase and received a detailed script describing the standardized way of interacting for each scenario (see table 1 (Tab. 1)). We rehearsed the simulation with advanced medical students and physicians in postgraduate training. Elderly SPs simulated all chest pain scenarios for a more realistic portrayal of a possible cardiac origin of chest pain. The elderly chest pain SPs were trained portraying acute coronary syndrome previously and received additional training for costosternal syndrome and gastrointestinal reflux. Male SPs exclusively portrayed the abdominal pain scenarios to exclude gynaecological differential diagnoses. SPs completed a four hours training, including a rehearsal for every scenario with house officers. 

Raters were General Practitioners (GPs) from the teaching practices network of the faculty. Most of them have been involved in rating OSCE for many years. All received a 15-30 minutes introduction to the new principles of the MS-OSCE before making their first assessment. The checklist for each chief complaint was identical. The scenarios were recapitulated with the SPs. Each station was assessed by 1 rater. During the three days of examination 23 raters were engaged. Two raters rated at all stations while most raters only rated at one or two stations.

Students enrolled electronically for a specific day and time slot. They were assigned to 2 groups of 5 students each. Two groups simultaneously circulated through a 5 stations course in a corridor with 10 separate rooms. The scenario to be simulated was randomly selected by the rater before the student entered the station. Students had 10 minutes at each station to complete the task and additional time to switch between stations. The entire MS-OSCE took 60 minutes for every student.

#### Assessment and grading

Federal regulations of examination in medical education in Germany require grading on an ordinal scale ranging from 1 to 5 (excellent (1), good (2), fair (3), sufficient (4) and fail (5)). This scale is used in a similar way in German schools and is familiar to all raters [https://www.gesetze-im-internet.de/_appro_2002/BJNR240500002.html]. We assessed students’ performance with a checklist (checklist rating (CR)), which consisted of either binary items (e.g. student asked about smoking: yes/no) or Likert scales (e.g. quality of student–patient interaction). Checklist-items covered an identical examination routine for each scenario of a chief-complaint. Items fulfilled by more than 90% or less than 10% of the students were eliminated post hoc from the checklist. Communication was assessed with the Berlin Global Rating Scale grade (BGR) [18], a global rating scale [19], [20] based on the rating scale introduced by Hodges [21], adapted and validated for German assessment needs. Finally, raters had to give their intuitive overall global rating (OGR) [22] of each student’s overall performance at each station. OGR is needed to calibrate CR and BGR for aspects that are not captured by the checklist. The final grade for each station was calculated as the mean of CR, BGR and OGR. According to the examination regulations at the University of Greifswald, a pre-fixed cut-off-score of 60% was set as standard for failure.

#### Statistical analysis

We display grades across scenarios as box-plots with average, median, interquartile range, and outliers. The internal consistency of the OSCE was assessed with Cronbach’s alpha, based on the grades at each station. 

We computed intraclass correlations (ICC) to express the fraction of variance of the grade due to scenarios or raters. Ideally the fraction should be close to zero. For this purpose we computed linear regression models separately for each station, using a bootstrap approach for variance estimation because of violations of the normal distributions of the residuals. We used two sets of predictors:

1. dummy coded scenarios and raters (see table 2 (Tab. 2));
2. the first model and additionally the mean grade from all stations other than the outcome station (see table 3 (Tab. 3)).

The grades were included to correct for students’ overall skills on all stations except for the station under study. Computations were conducted with the xtreg command in stata, using the fixed-effects estimator. There were no missing data for the assessed variables.

Analyses were conducted in Stata 13 (Stata Corp., College Station, TX).

## Results

Stations and raters as well as scenarios were statistically independent of each other (see attachment 1 and attachment 2 ). The internal consistency of the OSCE according to Cronbach’s alpha across the five grades for the stations was 0.65 (CI_90 one sided_ 0.59). 

### Comparison of the scenarios at each OSCE Station 

**Figure 1:**
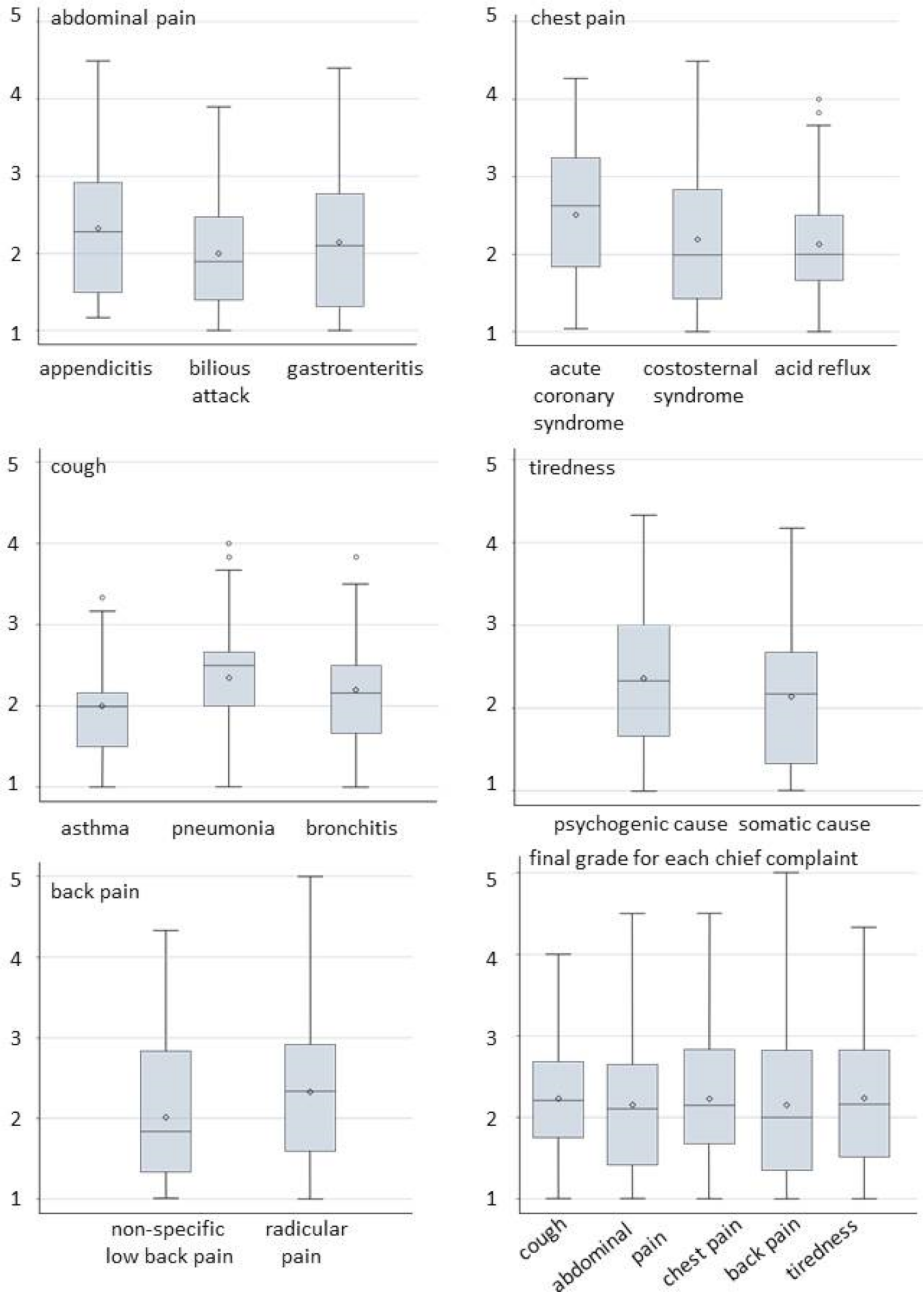
The distribution of the grades is displayed as box plots showing the median (horizontal line), average (diamond) and the interquartile range (lengths of the box). The vertical lines (whiskers) show minimum and maximum values excluding outliers, which are displayed as dots.

The distribution of grades for each scenario within stations and the distribution of final grades derived from the grades at each station are shown in figure 1 (Fig. 1). The average grade at each station ranged from 2.16 to 2.28. The difference of the average grade between the scenarios at each station ranged from 0.03 to 0.40 (see table 2 (Tab. 2) and table 3 (Tab. 3)). The largest difference was observed at the station assessing chest pain management. The life-threatening scenario ACS had a worse average grade of 0.4 compared to the scenario of gastrointestinal reflux. A similar moderately worse grade of 0.3 was observed for the scenario of appendicitis compared to gastroenteritis. The final grades for the chief complaints (stations) ranged from 1 to 5. 

#### Effect of scenarios and raters on the grades at each station

The effect of scenarios and raters on the grades at each station are expressed as ICCs and displayed in table 2 (Tab. 2) and table 3 (Tab. 3). We report the ICC unadjusted for students' skills (see table 2 (Tab. 2)) and the ICC adjusted for students’ skills at the other OSCE-stations (see table 3 (Tab. 3)). The effect of the scenarios on the grades at the stations ranged from 5.2% to 7.8% without taking mean grades at the other stations into account and adjusted from 4.2% to 9.2% when taking the mean grade into account. Corresponding to the largest difference in average grades between the scenarios, the largest effect of scenario was observed at the station assessing chest pain. 

The number of raters at each station varied from 6 to 10 over the three days. The unadjusted effect of the raters on the grades at the stations ranged from 14.1% to 39.8% without taking mean grades at the other stations into account and from 20.5% to 50.3% if doing so. The largest effect of raters was observed at the station assessing abdominal pain. 

## Discussion

### Summary of the main results

A total of 169 second clinical year students and 23 raters participated in the MS-OSCE. The difference of the mean grades between the scenarios of a given chief complaint ranged from 0.03 to 0.4 on a 1-5 grading scale. The effect of scenarios on students’ grades at a station accounted for 4% to 9% of the total variability of the grades, the respective figures for raters ranged from 20% to 50% adjusted for students’ skills. 

#### Meaning of the findings

We observed differences in the distribution of the final grades between the scenarios ranging from 0.03 to 0.4 on the 5-point rating system (see figure 1 (Fig. 1)). Although the checklist-items cover an identical examination routine for each scenario, rating should not be affected by the severity of the portrayed underlying diagnosis, since we expect students to explore all possibilities. It seems that missing the diagnosis or committing management errors for a potentially life-threatening scenario like ACS, appendicitis or pneumonia resulted in worse grades than similar mistakes with a corresponding benign scenario as costosternal syndrome, gastroenteritis, or bronchitis. There is no consensus what is considered a meaningful difference; we consider the observed difference as minor to moderate. 

Compared to the magnitude of the effect of different raters on the grades at a station the effect of the different scenario was small but still relevant. The effect of the raters was independent of the scenarios and students’ ability. The difference in the average grade awarded between the most lenient and strictest rater exceeded more than 1 grade on the 5-point rating scale, suggesting possibly poor inter-rater reliability. Therefore, calibrating raters seems to be far more important than adjusting for differences in the difficulty of scenarios. Wilkinson et al. [23] showed “that examiner factors contribute substantially more to the objectivity of an OSCE than do mark sheets or checklists”. Inter-rater reliability in OSCEs is rarely reported and varies according to OSCE construction, rating instrument used (global rating/checklist rating) and assessment conditions (direct observation/ video) [20], [24], [25]. Hatala et al. [26] piloted an OSCE with 2 stations, fragmented into 3 subsequent sequences of 10 minutes each to cover multiple content areas relevant to internal medicine. They observed an inter-rater reliability ranging from 0.63 to 0.91 with two raters for each scenario. Brennan and colleagues [16] found that although the range of grades awarded varied if examiners changed at OSCE stations (total number of raters at a given station not stated), examination reliability and the likely candidate outcome were not affected.

Due to financial constraints, we - like many other medical schools - cannot afford to assess each OSCE station with two raters simultaneously. 

More intensive training of raters and SPs [4] as well as a more thorough development of checklists to establish better inter-rater reliability are possible remedies to reduce the effect of raters on grading. However, the assumption that a more intensive rater training increases inter-rater reliability does not always hold true [27], [28]. Which amount of unfairness and lack of reliability should be accepted and to which degree the effect of raters can be reduced is a matter of debate [29]. 

We do not believe that MS-OSCE has reduced exchange of information, but we assume subjectively that the switch to MS-OSCE has led to a more complete history taking and physical examination and a less hasty performance throughout the whole 3 days of the annual OSCE. However, we have no objective measurement supporting this assumption. 

#### Strengths and limitations 

This is to our knowledge the first report of a MS-OSCE. We calculated the impact of multiple scenarios and raters on the grades in a MS-OSCE adjusting for students’ skills. We did not establish inter-rater correlations for the checklists and provided only minimal rater training, due to lack of resources. This reflects most likely the situation at many medical schools assessing students’ skills with OSCE. There was a good correlation ranging from 0.6 to 0.8 between the checklist rating and global rating (results not shown), indicating congruent ratings of communication and examination skills. We cannot exclude effects on students’ performance due to different accuracy in portrayal of scenarios by different SPs portraying the same scenario during three days of examination. We did not attempt to adjust for SPs. Additionally we did not investigate or adjust for gender effects which have been shown to effect grading [29], [30], [31]. Varying gender of SPs might have influenced students’ performance at the chest pain station and the acute cough station, where auscultation was within the scope of the demanded skills. Our MS-OSCE with only five stations is relatively short. It has been postulated that at least 10 stations are needed for a reliable assessment [32], [33]. Ten minutes per station is in an accepted time range [34], [35] and even high-stakes examinations demand only 15 minutes per OSCE-station for patient encounter [36]. We have a good internal consistency (Cronbach’s alpha: 0.65) over all stations compared with other reports from the literature [32]. 

Although it is possible to adjust students’ individual grades for differences in scenario and for differences between raters with a correction factor after taking the exam, we did not adjust accordingly.Calculation of correction factors after each exam would require resources which are currently not available to us. 

Validity measurements are not in the scope of our report. Van der Vleuten and Schuwirth [7] state that key issues concerning the validity of competence assessments are authenticity of performance and the integration of professional competencies. MS-OSCE addresses the authenticity of students’ performance by providing several scenarios at one station to reduce the effect of shared information (cheating) on students’ case management. Content validity was assured by reviewing MS-OSCE-stations by a team of experienced teaching physicians. Providing SP-based clinical scenarios at each station, assessment by standardised ratings (checklists) and a validated global rating instrument, face validity of the MS-OSCE might equal that of a traditional OSCE with only 5 stations. 

## Conclusions

The effect of different scenarios on the grades assessing the management of one chief complaint in General Practice was small compared to the effect of raters. Improving inter-rater reliability is more important to ensure objectivity and fairness of OSCE than providing the same scenario to all students. 

## List of abbreviations

ACS: acute coronary syndrome

BGR: Berlin Global Rating Scale 

CI: confidence interval

CR: checklist rating

GP: General Practitioner

OGR: overall global rating

ICC: intraclass correlation coefficient

MS-OSCE: Multiple Scenario Objective Structured Clinical Examination

OSCE: Objective Structured Clinical Examination

SP: Standardized Patient

## Acknowledgements

We are grateful to Francis Baudet, Gisela Greschniok, Heinz Hammermayer, Thomas Hannemann, Mathias Herberg, Gero Kärst, Andreas Krüger, Barbara Krüger, Annika Matz, Hans-Diether Seiboth, Thomas Richter, Claudia Runge, Carmina Spreemann, Antje Theurer, Renate Tilchner, Rüdiger Titze, Arne Wasmuth, Christine Wendt, Arno Wilfert.

## Data availability

Data is available on reasonable request.

## Authors’ contributions

JS and JFC conceived the multiple scenario OSCE, the scenarios and rating sheets were developed and piloted by JS, CR, GW, AA, FL, AH, JFC. CR, JS, FL and GW trained the simulation patients, AH was responsible for data management, COS was leading the statistical analysis. JS and JFC wrote the first draft which was revised and approved by all authors.

## Competing interests

The authors declare that they have no competing interests. 

## Supplementary Material

Independence of stations and raters. Information attachment 1: to assess independence Chi² or Fisher exact test was calculated.

Independence of scenarios. Information attachment 2: to assess independence Chi² or Fisher exact test was calculated.

## Figures and Tables

**Table 1:**
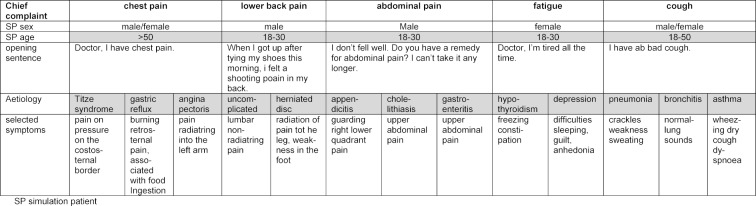
Chief complaints with matching scenarios

**Table 2:**
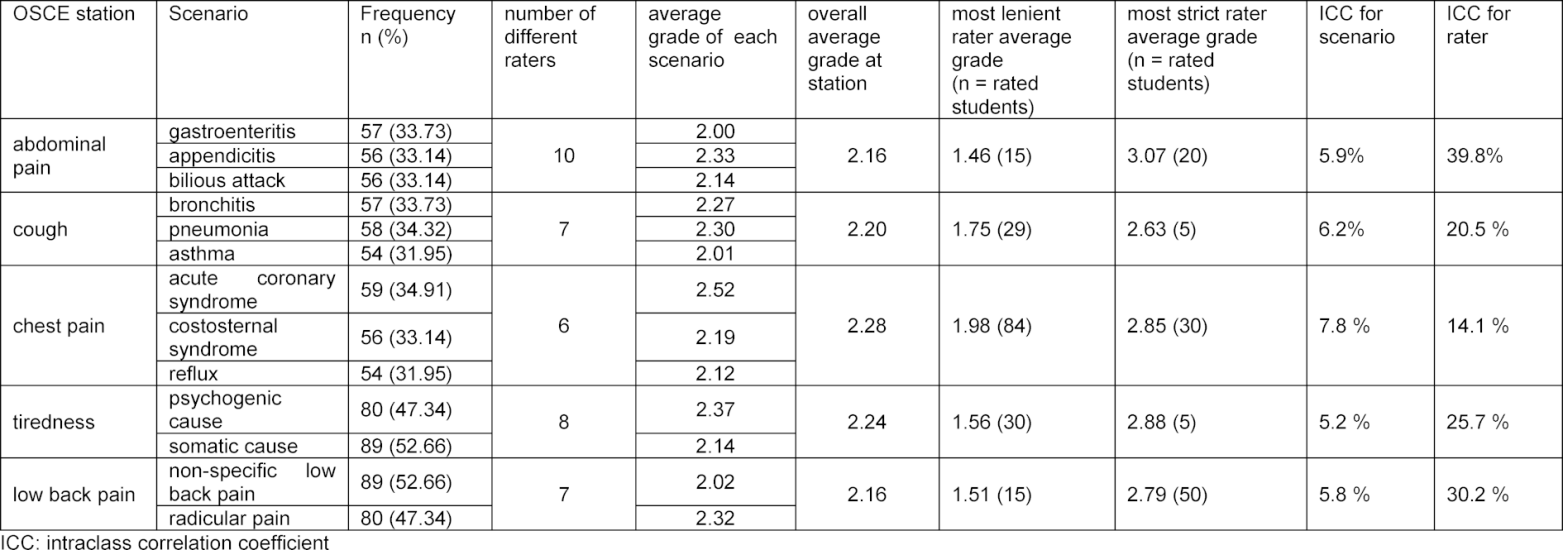
Effect of station and raters on the grade unadjusted for students’ skills

**Table 3:**
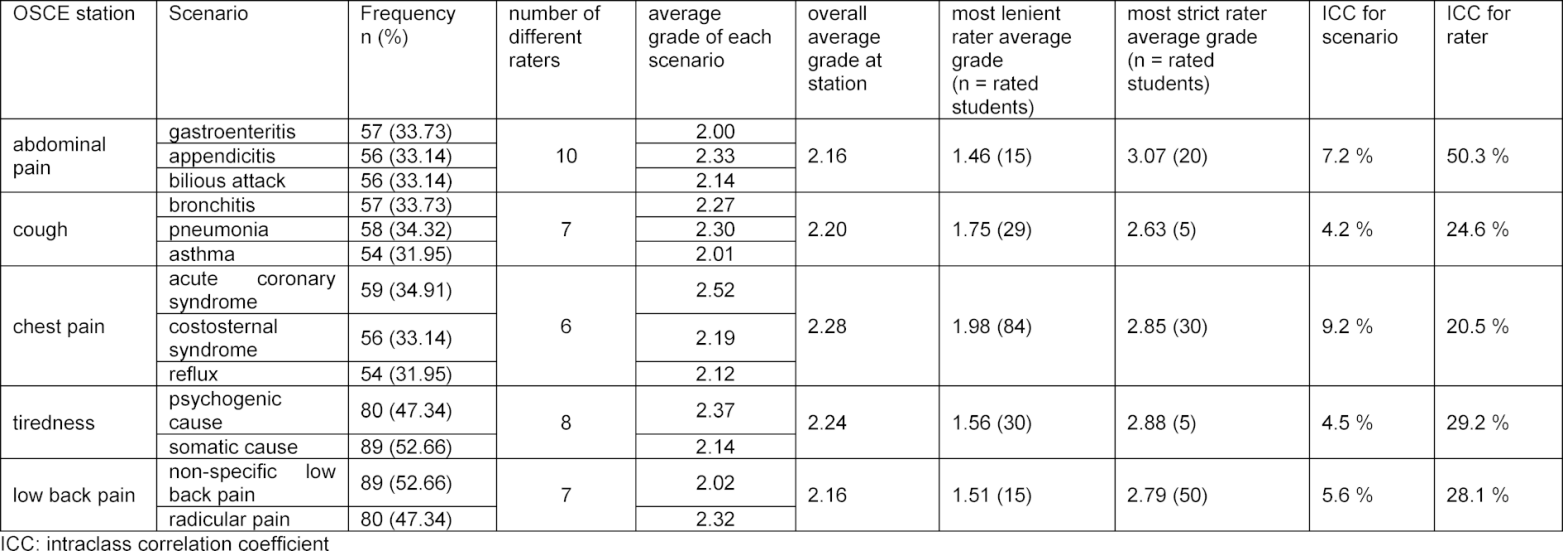
Effect of station and raters on the grade adjusted for students’ skills
