# Supplementary material for: Fairness and objectivity of a multiple scenario objective structured clinical examination
Source: GMS J Med Educ. 2019 May 16;36(3):Doc26. doi: 10.3205/zma001234 (PMC6545613; doi:10.3205/zma001234)
Supplement: Independence of stations and raters. Information attachment 1: to assess independence Chi² or Fisher exact test was calculated. [file JME-36-3-26-s-001.pdf]

. *** correlations stations and raters

abdominal pain_st	abdominal pain_rater 2101	2111	2112	2113	2114	2115	2116	2117	2118	2119	Total
scen 1	7	5	6	5	6	5	6	7	5	5	57
	6.7	5.1	6.7	5.1	5.1	5.1	6.7	6.7	4.7	5.1	57.0
scen 2	8	5	7	5	5	5	7	7	4	3	56
	6.6	5.0	6.6	5.0	5.0	5.0	6.6	6.6	4.6	5.0	56.0
scen 3	5	5	7	5	4	5	7	6	5	7	56
	6.6	5.0	6.6	5.0	5.0	5.0	6.6	6.6	4.6	5.0	56.0
Total	20	15	20	15	15	15	20	20	14	15	169
	20.0	15.0	20.0	15.0	15.0	15.0	20.0	20.0	14.0	15.0	169.0

_st : station
scen : scenario

Pearson chi2(18) = 3.1429 Pr = 1.000
Fisher's exact = 1.000

. tab cough_station cough_rater, chi2 exp exact

cough_st	cough_rater 2102	2115	2116	2120	2121	2122	2123	Total
scen 1	1	5	1	11	25	12	2	57
	2.4	5.1	1.7	9.8	26.6	9.8	1.7	57.0
scen 2	2	5	2	11	26	10	2	58
	2.4	5.1	1.7	10.0	27.1	10.0	1.7	58.0
scen 3	4	5	2	7	28	7	1	54
	2.2	4.8	1.6	9.3	25.2	9.3	1.6	54.0
Total	7	15	5	29	79	29	5	169
	7.0	15.0	5.0	29.0	79.0	29.0	5.0	169.0

Pearson chi2(12) = 5.3352 Pr = 0.946
Fisher's exact = 0.955

. tab chestpain_station chestpain_rater, chi2 exp exact

chest pain _st	chest pain_rater 2102	2103	2104	2115	2116	2118	Total
scen 1	30	7	2	5	5	10	59
	29.3	7.0	1.7	5.2	5.2	10.5	59.0
scen 2	27	7	2	5	5	10	56
	27.8	6.6	1.7	5.0	5.0	9.9	56.0
scen 3	27	6	1	5	5	10	54
	26.8	6.4	1.6	4.8	4.8	9.6	54.0
Total	84	20	5	15	15	30	169
	84.0	20.0	5.0	15.0	15.0	30.0	169.0

Pearson chi2(10) = 0.4974 Pr = 1.000
Fisher's exact = 1.000

. tab fatigue_station fatigue_rater, chi2 exp exact

fatigue_st	fatigue_rater 2104	2115	2116	2117	2123	2224	2225	2226	Total
scen 1	2	18	14	11	2	12	11	10	80
	2.4	16.6	14.2	11.4	2.4	11.8	11.8	9.5	80.0
scen 2	3	17	16	13	3	13	14	10	89
	2.6	18.4	15.8	12.6	2.6	13.2	13.2	10.5	89.0
Total	5	35	30	24	5	25	25	20	169
	5.0	35.0	30.0	24.0	5.0	25.0	25.0	20.0	169.0

Pearson chi2(7) = 0.6511 Pr = 0.999
Fisher's exact = 0.999

. tab back pain_station back pain_rater, chi2 exp exact

back pain _st	back pain_rater 2114	2115	2116	2123	2401	2402	2403	Total
scen 1	7	2	9	7	28	26	10	89
	7.9	2.6	7.9	7.9	25.8	26.3	10.5	89.0
scen 2	8	3	6	8	21	24	10	80
	7.1	2.4	7.1	7.1	23.2	23.7	9.5	80.0
Total	15	5	15	15	49	50	20	169
	15.0	5.0	15.0	15.0	49.0	50.0	20.0	169.0

Pearson chi2(6) = 1.5384 Pr = 0.957
Fisher's exact = 0.959

_st : station

scen : scenario

To assess independence Chi² or Fisher exact test was calculated.
