# Supplementary material for: Fairness and objectivity of a multiple scenario objective structured clinical examination
Source: GMS J Med Educ. 2019 May 16;36(3):Doc26. doi: 10.3205/zma001234 (PMC6545613; doi:10.3205/zma001234)
Supplement: Independence of scenarios. Information attachment 2: to assess independence Chi² or Fisher exact test was calculated. [file JME-36-3-26-s-002.pdf]

abdominal pain_st	cough_st			
	scen 1	scen 2	scen 3	Total
scen 1	19	19	19	57
	19.2	19.6	18.2	57.0
scen 2	21	18	17	56
	18.9	19.2	17.9	56.0
scen 3	17	21	18	56
	18.9	19.2	17.9	56.0
Total	57	58	54	169
	57.0	58.0	54.0	169.0

Pearson chi2(4) = 0.7653 Pr = 0.943

abdominal pain_st	fatigue_st		
	scen 1	scen 2	Total
scen 1	30	27	57
	27.0	30.0	57.0
scen 2	26	30	56
	26.5	29.5	56.0
scen 3	24	32	56
	26.5	29.5	56.0
Total	80	89	169
	80.0	89.0	169.0

Pearson chi2(2) = 1.1103 Pr = 0.574

abdominal pain_st	back pain_st		
	scen 1	scen 2	Total
scen 1	33	24	57
	30.0	27.0	57.0
scen 2	26	30	56
	29.5	26.5	56.0
scen 3	30	26	56
	29.5	26.5	56.0
Total	89	80	169
	89.0	80.0	169.0

Pearson chi2(2) = 1.5175 Pr = 0.468

fatigue_st	chest pain_st			
	scen 1	scen 2	scen 3	Total
1	28	25	27	80
	27.9	26.5	25.6	80.0
2	31	31	27	89
	31.1	29.5	28.4	89.0
Total	59	56	54	169
	59.0	56.0	54.0	169.0

Pearson chi2(2) = 0.3170 Pr = 0.853

back pain _st	abdominal pain_st			
	scen 1	scen 2	scen 3	Total
scen 1	33	26	30	89
	30.0	29.5	29.5	89.0
scen 2	24	30	26	80
	27.0	26.5	26.5	80.0
Total	57	56	56	169
	57.0	56.0	56.0	169.0

Pearson chi2(2) = 1.5175 Pr = 0.468

back pain _st	cough_st			
	scen 1	scen 2	scen 3	Total
scen 1	31	32	26	89
	30.0	30.5	28.4	89.0
scen 2	26	26	28	80
	27.0	27.5	25.6	80.0
Total	57	58	54	169
	57.0	58.0	54.0	169.0

Pearson chi2(2) = 0.6559 Pr = 0.720

cough_st	abdominal pain_st			
	scen 1	scen 2	scen 3	Total
scen 1	19	21	17	57
	19.2	18.9	18.9	57.0
scen 2	19	18	21	58
	19.6	19.2	19.2	58.0
scen 3	19	17	18	54
	18.2	17.9	17.9	54.0
Total	57	56	56	169
	57.0	56.0	56.0	169.0

Pearson chi2(4) = 0.7653 Pr = 0.943

cough_st	chest pain_st			
	scen 1	scen 2	scen 3	Total
scenario 1	25	19	13	57
	19.9	18.9	18.2	57.0
scenario 2	17	20	21	58
	20.2	19.2	18.5	58.0
scenario 3	17	17	20	54
	18.9	17.9	17.3	54.0
Total	59	56	54	169
	59.0	56.0	54.0	169.0

Pearson chi2(4) = 4.3450 Pr = 0.361

cough_st	fatigue_st		
	scen 1	scen 2	Total
scen 1	29	28	57
	27.0	30.0	57.0
scen 2	30	28	58
	27.5	30.5	58.0
scen 3	21	33	54
	25.6	28.4	54.0
Total	80	89	169
	80.0	89.0	169.0

Pearson chi2(2) = 2.2804 Pr = 0.320

cough_st	back pain_st		
	scen 1	scen 2	Total
scen 1	31	26	57
	30.0	27.0	57.0
scen 2	32	26	58
	30.5	27.5	58.0
scen 3	26	28	54
	28.4	25.6	54.0
Total	89	80	169
	89.0	80.0	169.0

Pearson chi2(2) = 0.6559 Pr = 0.720

chest pain _st	cough_st			
	scen 1	scen 2	scen 3	Total
scen 1	25	17	17	59
	19.9	20.2	18.9	59.0
scen 2	19	20	17	56
	18.9	19.2	17.9	56.0
scen 3	13	21	20	54
	18.2	18.5	17.3	54.0
Total	57	58	54	169
	57.0	58.0	54.0	169.0

Pearson chi2(4) = 4.3450 Pr = 0.361

chest pain _st	fatigue_st		
	scen 1	scen 2	Total
scen 1	28	31	59
	27.9	31.1	59.0
scen 2	25	31	56
	26.5	29.5	56.0
scen 3	27	27	54
	25.6	28.4	54.0
Total	80	89	169
	80.0	89.0	169.0

Pearson chi2(2) = 0.3170 Pr = 0.853

chest pain _st	back pain_st		
	scen 1	scen 2	Total
scen 1	32	27	59
	31.1	27.9	59.0
scen 2	30	26	56
	29.5	26.5	56.0
scen 3	27	27	54
	28.4	25.6	54.0
Total	89	80	169
	89.0	80.0	169.0

Pearson chi2(2) = 0.2308 Pr = 0.891

fatigue_st	abdominal pain_st			
	scen 1	scen 2	scen 3	Total
scen 1	30	26	24	80
	27.0	26.5	26.5	80.0
scen 2	27	30	32	89
	30.0	29.5	29.5	89.0
Total	57	56	56	169
	57.0	56.0	56.0	169.0

Pearson chi2(2) = 1.1103 Pr = 0.574

fatigue_st	cough_st			
	scen 1	scen 2	scen 3	Total
scen 1	29	30	21	80
	27.0	27.5	25.6	80.0
scen 2	28	28	33	89
	30.0	30.5	28.4	89.0
Total	57	58	54	169
	57.0	58.0	54.0	169.0

Pearson chi2(2) = 2.2804 Pr = 0.320

back pain _st	chest pain_st			
	scen 1	scen 2	scen 3	Total
scen 1	32	30	27	89
	31.1	29.5	28.4	89.0
scen 2	27	26	27	80
	27.9	26.5	25.6	80.0
Total	59	56	54	169
	59.0	56.0	54.0	169.0

Pearson chi2(2) = 0.2308 Pr = 0.891

_st : station

scen : scenario

To assess independence Chi^2 or Fisher exact test was calculated.
